# Supplementary material for: The estimated benefits of increasing cigarette prices through taxation on the burden of disease and economic burden of smoking in Nigeria: A modeling study
Source: PLoS One. 2022 Mar 2;17(3):e0264757. doi: 10.1371/journal.pone.0264757 (PMC8890735; doi:10.1371/journal.pone.0264757)
Supplement: Attachment — Submitted filename: Review.docx [file pone.0264757.s004.docx]

This paper estimates the benefits of increasing cigarette prices through taxation on the burden of disease and economic burden of smoking in Nigeria. It is based on a widely used and published microsimulation model. This paper can prove very useful to inform tobacco control policy decisions in Nigeria, specifically tobacco tax increases. The manuscript would, however, benefit from significant revision in clarifying the methods of analysis and presenting the results. I have made section-specific comments and suggestions as follows to help improve the paper.

**Introduction**

1. Page 3 lines 44-45: The Introduction starts with the citation of the tobacco-attributable deaths and disabilities for 2017. More recent estimates of tobacco-attributable deaths and disabilities are available for 2019. Please see the quote below:

“Globally in 2019, smoking tobacco use accounted for 7·69 million (7·16–8·20) deaths and 200 million (185–214) disability-adjusted life-years, and was the leading risk factor for death among males (20·2% [19·3–21·1] of male deaths). 6·68 million [86·9%] of 7·69 million deaths attributable to smoking tobacco use were among current smokers.”

Reference: GBD 2019 Tobacco Collaborators. Spatial, temporal, and demographic patterns in prevalence of smoking tobacco use and attributable disease burden in 204 countries and territories, 1990–2019: a systematic analysis from the Global Burden of Disease Study 2019. Lancet 2021; 397: 2337–60 Published Online May 27, 2021 https://doi.org/10.1016/ S0140-6736(21)01169-7.

2. Page 4 line 70: In the cost-of-illness approach to the estimation of the economic burden of tobacco use, the direct medical costs include the treatment costs incurred by both the public health system and the out-of-pocket expenses of the patients and their families. By accounting the costs of “treatment incurred on the health system” only, this paper underestimates the direct medical costs. Please clarify how the out-of-pocket expenses that generally account for a major fraction of total medical expenditures were considered.

**Methods**

1. Page 4 lines 92-94: “The risk of acute and chronic events is estimated from 93 the baseline risk in non-smokers multiplied by the age, gender, and condition-specific relative 94 risks (RR) for smokers and ex-smokers.” Based on the citation (14), it seems that the authors used the RRs from a study in the U.S. Please explain why the U.S. based RRs were used and how these estimates were validated for Nigeria. I would recommend the authors use evidence available from countries that are comparable to Nigeria or at least from the same region. They can justify the use of U.S. estimates if none of these estimates are available and if the previous studies based on the same model, such as citations (8-13), used the same parameters.

2. Page 5 lines 102-105: Please use a reference and the ICD-10 codes for the list of health conditions considered for the study.

3. Page 5 lines 106-110: The extrapolation of the U.S. estimates of the consequences of passive smoking to Nigeria may be far off the realities in Nigeria given the wide differences in population characteristics, disease events, and healthcare systems in the two countries. Similar to Comment 1 on Methods, I would recommend the authors use evidence available from countries that are comparable to Nigeria or at least from the same region. They can justify the use of U.S. estimates if none of these estimates are available and if the previous studies based on the same model, such as citations (8-13), used the same parameters.

4. Page 6 lines 129-130: It is not clear how the variation in smoking prevalence (page 5 line 121) was translated into variation in consumption to estimate the effect on tax revenue.

5. In page 7 lines 155, 158, 159 and un Table 2, the notation of the local currency is different (₦ or NGN). The Results section in the Abstract, on the other hand, mentions Naira. Please make it consistent across the manuscript. When it is used the first time in the manuscript, it should be mentioned that it is Nigerian local currency Naira.

6. Page 10 lines 207-210: The household expenditure needs to be converted to per capita household expenditure by dividing total expenditure by household size to be assigned to individuals’ annual income. It is not clear whether this conversion was done or not.

7. Page 10 lines 210-213: IMF provide projection of per capita GDP for 4/5 years from current year when the World Economic Outlook data is updated each year. These projections are based on models that consider several growth factors and their recent trends. I would recommend authors use the growth rate projected in these data instead of using mean of annual growth rates from 1960 to 2019 which may be biased due to the long horizon of 60 years when many low- and middle-income countries have made major strides in economic growth.

8. Page 10 line 220: What does the term “profit associated with the disease” imply? Please clarify.

**Results**

1. How do the deaths and events attributable to tobacco use estimated in this study compare the estimates available in the Global Burden of Disease Study? These can be compared to show the validity of the study findings.

2. In Table 3, the last two columns present % of attributable cost whereas the head of the column state “Smoking-attributable costs (millions)”. Please consider revising the head.

3. Table 3 would be more legible in landscape page orientation and broken into two pages.

4. The two columns reporting Economic burden (in millions) in Table 3 are not discussed in the text. The title of the table says, “direct costs for the healthcare system”. So, I am assuming these columns refer to the medical costs. But the economic burden implies indirect costs or the productivity losses as well. Are the indirect costs included in these estimates? If yes, then the title needs to be revised accordingly.

5. Page 13 lines 256-258: The estimation of the burden of passive smoking is explained in the Methods section. But the burden of perinatal disease and accidents related to smoking is mentioned for the first time in the paper in the Results section. How were these estimates obtained?

6. Page 14 lines 264-276: The costs estimates reported in the section on Economic Burden do not match the economic burden estimates presented in Table 3. It seems these estimates are presented in Table 5. Please ensure the correct and consistent reporting of the cost estimates.

7. The estimates in Table 5 refer to 10-year effects. It is not clearly mentioned in the Methods section that the simulation was done for the 10-year period following the tax increase.

8. Table 5 reports the effects of price increases by 25%, 50% and 75% and discusses the results of only 50% price increases. It is not clear why the authors wanted to include the estimates of the effects of 25% and 75% price increases and hence those estimates seem redundant. If they are keen on keeping these estimates, I would recommend making the point that higher price increases would lead to greater cost saving and revenue gain.

9. Page 16 lines 306-312: The authors refer to the global study Goodchild et al to present the global estimates. How do the cost estimates for Nigeria in this global study (presented in the Supplementary Material) compare to the estimates obtained in the present study?

**Discussion**

1. Page 18 lines 354-357: This paragraph reporting amount of tax increase by 168% leading to 50% price increase and 101% increase in fiscal revenue belongs to the Results section.

2. Page 18 lines 364-366: Is the comparison of direct cost in Nigeria to the average of LA countries in absolute levels? What is the unit of this measurement? For cross-country comparison, the estimates should be converted to per capita terms and PPP dollars to adjust for population size and differences in purchasing power of dollars.

3. Page 10 lines 400-402: This paper does not estimate the net economic benefits. Because there is a cost of implementation of tobacco tax and the net benefit should exclude this cost that was not taken into consideration. I suggest dropping the part “resulting in substantial net economic benefits for Nigeria” from the concluding statement.

**Supporting Information**

The data sources for the tables in the Appendices S1, S2 and S3 should reported with full citation.
